# Molecular epidemiology of environmental MRSA at an equine teaching hospital: introduction, circulation and maintenance

**DOI:** 10.1186/1297-9716-45-31

**Published:** 2014-03-19

**Authors:** Joany van Balen, Jade Mowery, Micha Piraino-Sandoval, Rocio C Nava-Hoet, Catherine Kohn, Armando E Hoet

**Affiliations:** 1 Department of Veterinary Preventive Medicine, College of Veterinary Medicine, The Ohio State University, 1900 Coffey Road, Columbus, OH 43210, USA; 2 Veterinary Public Health Program, College of Public Health, The Ohio State University, 1900 Coffey Road, Columbus, OH 43210, USA; 3 Department of Veterinary Clinical Sciences, College of Veterinary Medicine, The Ohio State University, 1900 Coffey Road, Columbus, OH 43210, USA

## Abstract

The role that environmental contamination might play as a reservoir and a possible source of Methicillin-resistant *Staphylococcus aureus* (MRSA) for patients and personnel at equine veterinary hospitals remains undefined, as the environment has only been monitored during outbreaks or for short periods. Therefore, the objectives of this study were to determine the monthly presence, distribution, and characteristics of environmental MRSA at an equine hospital, and to establish patterns of contamination over time using molecular epidemiological analyses. For this purpose, a yearlong active MRSA surveillance was performed targeting the environment and incoming patients. Antimicrobial susceptibility testing, SCC*mec* typing, PFGE typing, and dendrographic analysis were used to characterize and analyze these isolates. Overall, 8.6% of the surfaces and 5.8% of the horses sampled were positive for MRSA. The most common contaminated surfaces were: computers, feed-water buckets, and surgery tables-mats. Ninety percent of the isolates carried SCC*mec* type IV, and 62.0% were classified as USA500. Molecular analysis showed that new pulsotypes were constantly introduced into the hospital throughout the year. However, maintenance of strains in the environment was also observed when unique clones were detected for 2 consecutive months on the same surfaces. Additionally, pulsotypes were circulating throughout several areas and different contact surfaces of the hospital. Based on these results, it is evident that MRSA is constantly introduced and frequently found in the equine hospital environment, and that some contact surfaces could act as “hot-spots”. These contaminated surfaces should be actively targeted for strict cleaning and disinfection as well as regular monitoring.

## Introduction

Methicillin-resistant *Staphylococcus aureus* (MRSA) is a widely spread opportunistic pathogen that has been found circulating on horse farms with prevalences ranging from 0.6% up to 4.7%; these horses are typically colonized with MRSA without manifesting clinical signs [1-6]. In contrast, a higher prevalence, from 5.8% to 12.0%, has been reported in horses admitted to veterinary hospitals, where the horses are more likely to manifest clinical illnesses mostly associated with joint, skin, traumatic wounds and surgical site infections, among others [7-11]. This pathogen has been isolated from horses worldwide, and the genotypic characteristics of MRSA strains found in the equine population vary within regions [1,3,5,8,10,12,13]. Moreover, certain clones like the well-known USA500, seems to be one of the most prevalent among equidae, and even the emerging LA-MRSA (ST398) has been recently described in horse populations [13-15].

In recent years, MRSA has become one of the most important nosocomial pathogens affecting equine hospitals. An example of this nosocomial transmission and impact was described in The Netherlands [16], where 33.9% (21/62) of all MRSA equine clinical cases that were managed at a veterinary teaching hospital (VTH) were caused by hospital acquired infections. These horses tested negative for MRSA at their time of arrival and acquired this pathogen during their hospitalization.

When studying the possible sources of MRSA in a veterinary hospital setting, two different scenarios could be considered as potential sources of this bacterium in horses: endogenous and exogenous. An endogenous source refers to animals that are already colonized at their time of arrival and are capable of self-inoculating themselves after a diagnostic or surgical procedure. In contrast, an exogenous source denotes the participation of an external component (human, animal or environment) as the plausible origin of this pathogen. This last scenario has been reported multiple times in which colonized or infected hospital personnel or contaminated hospital environments have been associated with the transmission of MRSA to horses in veterinary settings [8,17,18].

Environmental contamination has been considered a possible source of nosocomial MRSA infections that have occurred in equine hospitals [8,17,19]. MRSA is capable of surviving up to seven months on inanimate objects and contact surfaces of healthcare facilities [20]. Despite these facts, the presence of MRSA in equine hospital environments has been studied only during outbreak investigations or for very short periods of time [16,17,21,22]. As a result, the role that contaminated surfaces might play as a reservoir and a possible source of MRSA for patients and hospital personnel, as well as the type of strains frequently circulating in equine hospitals remains undefined. Therefore, the objectives of this study were to determine the presence and distribution of MRSA environmental contamination during one year at an equine teaching hospital; to determine the phenotypic and genotypic characteristics of the MRSA strains circulating in the hospital; as well as to establish patterns and changes of this contamination over time using molecular epidemiological analyses.

## Materials and methods

### Active MRSA surveillance

This study was conducted at the Galbreath Equine Center (GEC) from The Ohio State University Veterinary Medical Center over a one year period, between September 2009 and August 2010. The GEC is a large tertiary healthcare hospital that receives over 1700 equine patients per year, and provides services to the Ohio equine community as well as referral cases from private practices throughout the Midwestern US.

### Environmental locations, surfaces and sample collection method

Sixty seven environmental samples were collected from the GEC every month from the following services: Internal Medicine (16 samples/month), Intensive Care Unit (14 samples/month) and Surgery (26 samples/month). Samples were also collected from common areas that did not belong to any particular service, and were characterized as General Areas (11 samples/month). The number and locations of the surfaces to be sampled were determined based on the results obtained during a 2007 pilot study performed at GEC [21]. Furthermore, these areas were targeted, because MRSA found in any of these environments would represent a potential nosocomial risk for patients using these services.

Surfaces from the targeted services were categorized as human contact and animal contact surfaces (Table 1) as previously described [23]. Since the majority of the spaces around the GEC are very open and large in size, it would have been nearly impossible to individually sample all the surfaces of every room. Therefore, in some cases several surfaces in the same section/room were sampled as a pool (referred as Pool A to Q) to be able to cover as much area of the hospital as possible (see Table 1). All areas/rooms of GEC are due to be clean and disinfected at the end of the day, and general equipment (e.g. endoscope, feed & water buckets, surgery tables & mats) are required to be washed and disinfected between each patient. Environmental samplings were performed in late hours of the afternoon, before any cleaning was done by the hospital staff. In the case of the equipment, samples were collected regardless of the last time they were used and/or disinfected.

**Table 1:** Contact surfaces sampled with electrostatic cloths (■) or sterile swabs (▲) at an equine teaching hospital

**Hospital service**	**Human contact**	**Animal contact**
***Internal medicine*** (Triage room, Med room, ward, and aisles)	Twitches - handle ^■1^	Stocks ^■^
	Computers^▲2^	Railing ^■^
	Counter tops & cabinets ^■^	Floor, drain & stall mat ^■3^
	Doors ^■^	Twitches - chain ^▲1^
	Ultrasound - controls ^▲^	Ultrasound - probe ^▲^
	Endoscope - controls ^■^	Endoscope ^■^
	Pool A (Triage) ^■4^	Pool O (Ward) ^■5^
***Intensive care unit*** (Ward, ICU Radiology, ICU office, and aisles)	Doors ^■^	Feed & water buckets ^■1^
	Carts ^■^	Foal bed ^■^
	Charts & files ^■^	Foal cart ^■^
	Computers ^▲2^	Floor, drain & stall mat ^■3^
	Supply cart ^■^	Foal watch mats ^■^
	Pool B (Aisles) ^■4^	Pool P (Ward) ^■5^
	Pool C (Aisles) ^■6^	
***Surgery*** (Scrub room, Prep room, Surgery suites, Recovery rooms, and Orthopedic ward)	Doors ^■^	Stocks ^■^
	Counter tops & cabinets ^■^	Surgery table & mats ^■^
	Hoist controls ^▲^	Recovery – mats & floor ^■^
	Pool D (Suite B) ^■4^	Pool Q (Ward) ^■5^
	Pool E (Suite C) ^■4^	
	Pool F (Suite E) ^■4^	
	Pool G (Ward) ^■6^	
	Pool H (Scrub R.) ^■6^	
	Pool I (Prep R.) ^■6^	
***General areas*** (Treadmill room, Breezeway, Office/front desk, Isolation stalls, Milk room, LA Radiology and CT room)	Counter tops ^■^	Door, wall & floor ^■7^
	Doors ^■^	Floor ^■^
	Counter tops, cabinets & sink ^■7^	CT table ^■^
	Pool J (Breezeway) ^■8^	
	Pool K (Milk R.) ^■8^	
	Pool L (Treadmill) ^■6^	
	Pool M (LA radiology) ^■6^	
	Pool N (CT room) ^■6^	

^1^Three units of this type of equipment were sampled as a pool.

^2^Two computers (keyboards and mouse) were sampled as a pool.

^3^Surfaces sampled in three stalls as a pool.

^4^Pool samples A, B, D, E, and F included: Light switches, phone, oxygen and suction valves, radio, microwave, lamps and medical carts within the same area or room.

^5^Pool samples O, P, and Q included: Halters, hay bags, and muzzles (4 of each) within the same ward.

^6^Pool samples C, G, H, I, L, M, and N included: Counter tops, cabinets and other contact surfaces within the same area or room.

^7^Surfaces sampled from four isolation stalls as a pool.

^8^Pool samples J and K included: Light switches, counter tops, computers (keyboards and mouse), phone and doors within the same area or room.

Every month the same pre-selected surfaces were sampled using dry electrostatic cloths (for large surfaces) and sterile pre-moistened cotton swabs (for smaller surfaces) [23]. In the case of pooled samples, the same electrostatic cloth was used to consistently sample all the surfaces included in the pool. The size and location of the area sampled from each surface were always the same each month. If during the sampling date a pre-selected surface was not available (i.e. the endoscope), it was skipped until the next month. In the case of equipment with numerous units present at the hospital (e.g. twitches, feed & water buckets, and surfaces included in pooled samples O, P and Q), only 3 units were sampled as a pool from those available at the time of sampling. All collected samples were processed at the Diagnostic and Research Laboratory for Infectious Diseases (DRLID) at the OSU, College of Veterinary Medicine. Upon arrival in the laboratory, electrostatic cloths and swabs were placed in pre-enrichment media and incubated at 35 °C for 24 h [23].

### Source of equine isolates

To determine the potential role that incoming horses could have in introducing MRSA strains into the hospital, parallel to the monthly environmental surveillance, a convenience sample of equines admitted to the same targeted services of the hospital was performed. Upon arriving to the hospital, a signed consent form was obtained from the horse’s owner. Before any clinical examination was performed on the animal by the hospital personnel, samples were collected from three to four anatomical locations on each horse. These locations were the nares (both sides), armpits (both sides), perianal area, and skin lesions (if any were present). Samples were collected with sterile pre-moistened cotton swabs in TSB (BD BBL™ Trypticase Soy Broth, Becton, Dickinson and Company, Sparks, USA) and were kept at room temperature and away from direct light, until the end of the day when all collected samples were taken to DRLID for further processing. The procedures used to sample the horses were approved by the IACUC (Protocol Number 2010A00000099-R1). In addition to the equine isolates collected during the active surveillance, two specimens from post-surgical MRSA infections that occurred during the study period were also included. Neither one of these horses were sampled at their arrival. These clinical cases were diagnosed by the Clinical Microbiology Laboratory of the OSU Veterinary Medical Center and banked as part of the center’s routine passive surveillance.

### Bacterial isolation and characterization

Specimen screening was performed as described before [23] using selective and non-selective media. Identification of *S. aureus* colonies was achieved based on colony morphology and reactions to biochemical tests (mannitol fermentation, gram stain, catalase, tube coagulase, latex agglutination (Sure-Vue® Color Staph ID, Biokit USA, inc, Lexington, MA, USA), anillin fermentation, Polymyxin B susceptibility and acetoin production (Vogues-Proskauer test)). Growth on Oxacillin Screen Agar® (OSA) plates that contained 6 μg/mL of Oxacillin supplemented with NaCl (BD BBL™, Becton Dickinson and Company, Maryland, USA) were used to phenotypically classify isolates as methicillin-susceptible *S. aureus* (MSSA) or MRSA following the Clinical Laboratory Standards Institute protocols [24].

### Phenotyping

Antimicrobial susceptibility profiles of 110 environmental isolates were determined by testing against 15 antimicrobials drugs (Amikacin 30 μg, Ampicillin 10 μg, Amoxicillin with Clavulanic Acid 30 μg, Cefpodoxime 10 μg, Cephalothin 30 μg, Chloramphenicol 30 μg, Ciprofloxacin 2 μg, Clindamycin 2 μg, Doxycycline 30 μg, Enrofloxacin 5 μg, Erythromycin 15 μg, Gentamicin 1 μg, Oxacillin 1 μg, Sulfamethoxazole with Trimethoprim 25 μg and Tetracycline 30 μg) using the Kirby-Bauer Disc Diffusion technique following protocols described by CLSI [24]. In addition, Vancomycin resistance was assessed using Vancomycin Screen Agar plates (6 mg/L) (BD BBL™ Vancomycin Screen Agar, Dickinson and Company, Sparks, USA). Inducible Clindamycin resistance was tested using the D-test [25]. Throughout the text, the term multidrug resistant (MDR) will be used for isolates resistant to 3 or more antimicrobial classes (including beta-lactams after *mecA* gene confirmation).

### Genotyping: *mecA* gene confirmation, Staphylococcal Chromosomal Cassette (SCC) *mec* Characterization and Pulsed Field Gel Electrophoresis (PFGE)

Only 71 environmental isolates with unique phenotypic profiles were further characterized. All molecular techniques were performed as previously described [23]. Briefly, SCC*mec* typing (type I to type VI) and confirmation of the presence of the *mecA* gene were assessed using a modified version of a multiplex PCR [26]. All primer concentrations were adjusted by doubling the original concentration. PCR mixture contained 2 μL of DNA template and 12.5 μL of 2× Multiplex PCR Master Mix (Qiagen®, Foster City, CA, USA). Primers and molecular grade water were added to reach a final volume of 25 μL per reaction. A Gradient Thermocycler (Eppendorf, Hamburg, Germany) was used with the following cycling conditions: 95 °C for 15 min; 35 cycles of 94 °C for 30 s, 57 °C for 90 s and 72 °C for 90 s; and a final extension of 72 °C for 10 min. Seakem LE (Cambrex, Rockland, ME, USA) 3% agarose gels with 1× Tris-acetate-EDTA buffer were used to resolved PCR products with running conditions at 100 Volts for 2 h. Gels were visualized with ethidium bromide.

Macrorestriction digestion of genomic DNA was performed using the enzyme *SmaI*, following PFGE protocols established by the Centers for Disease Control and Prevention [27]. *Salmonella* serotype Branderup strain H9812 was digested with *Xba*I and used as a molecular size marker. A CHEF mapper system (Bio-Rad Laboratories, Nazareth, Belgium) was used to separate DNA fragments, and band patterns were analyzed using BioNumerics® software (version 6.6, Applied Maths, Ghent, Belgium). Dice coefficient and Unweighted Pair Group Method using Arithmetic averages (UPGMA) with 1% tolerance allowed the construction of dendrograms to establish relatedness between strains. Three different dendrograms were created; one including only environmental isolates, one containing only equine isolates (both incoming patients and clinical cases), and one with both environmental and equine isolates. Band patterns with ≥ 98% similarity were characterized as the same pulsotype. Groups of closely related pulsotypes with ≥ 80% similarity were classified as clusters. A CDC database containing 100 *S. aureus* strains with the most typical band patterns for each USA type was used to compare and characterize the environmental isolates (cutoff point of ≥ 80% similarity).

### Statistical analysis

Comparisons between types of contact surfaces (human vs. animal) and between services (internal medicine, intensive care unit, surgery and general areas) were performed by calculating Chi-square coefficients. Similarly, seasonality was evaluated by comparing results of four groups: data collected from January to March, April to June, July to September, and October to December. Chi-square coefficients were calculated using the statistical software STATA (Small Stata 12.0, StataCorp LP, Texas, USA), and logistic regression models were created (Proc GLIMMIX in SAS version 9.3, SAS Institute Inc, North Carolina, USA) using Turkey-Kramer method for multiple pairwise comparisons. Relationships were considered significant when their *P*-value was ≤ 0.05.

## Results

### General prevalence and characterization of environmental isolates

**Figure 1:**
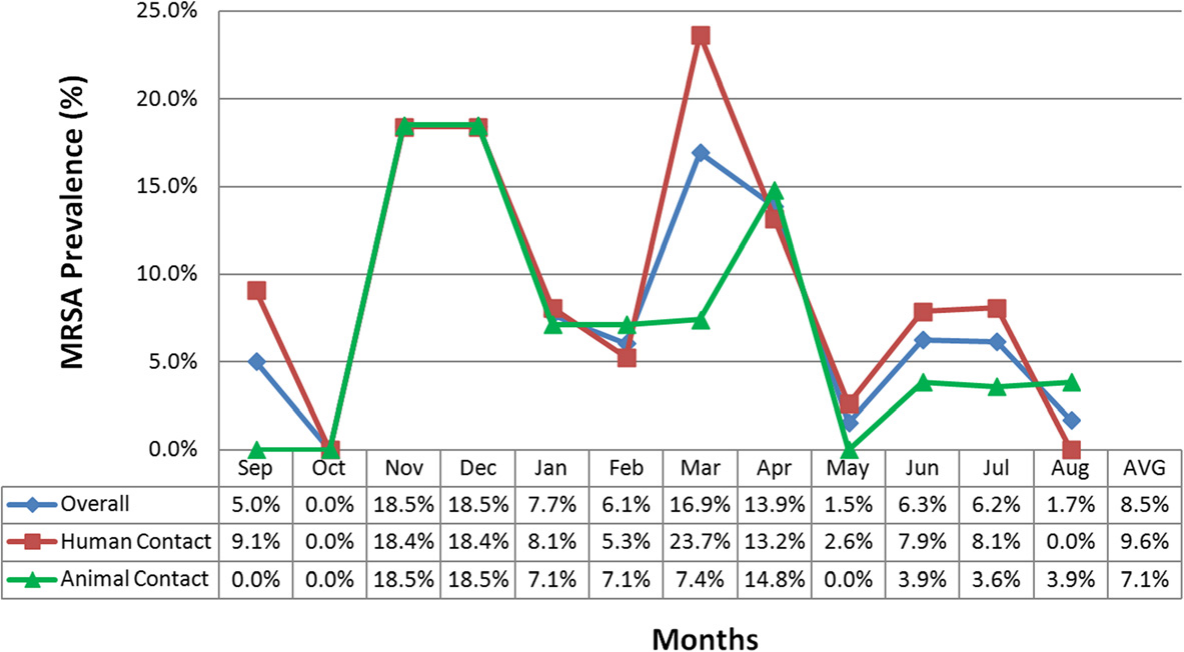
**Monthly distribution of environmental MRSA prevalence during one-year of active surveillance at an equine hospital.** For each month of the surveillance, prevalence’s for the overall (blue line), human contact surfaces (red line) and animal contact surfaces (green line) MRSA contamination are shown. The last column of the table represents the average (AVG) prevalence during the full year.

A total of 770 environmental samples were collected during the year long active surveillance. As it was explained before, if during the sampling date a pre-selected surface was not available it was skipped until the next month; therefore, a total of 34 surfaces were skipped due to this reason. During the twelve month period, 8.6% (66/770) of the overall surfaces sampled were found positive for MRSA. On a monthly basis, MRSA contamination ranged from 0.0% to 18.5% (Figure 1). Of the 66 positive surfaces, 71 unique isolates were obtained, indicating that some surfaces were contaminated with two different isolates at the moment they were sampled.

**Figure 2:**
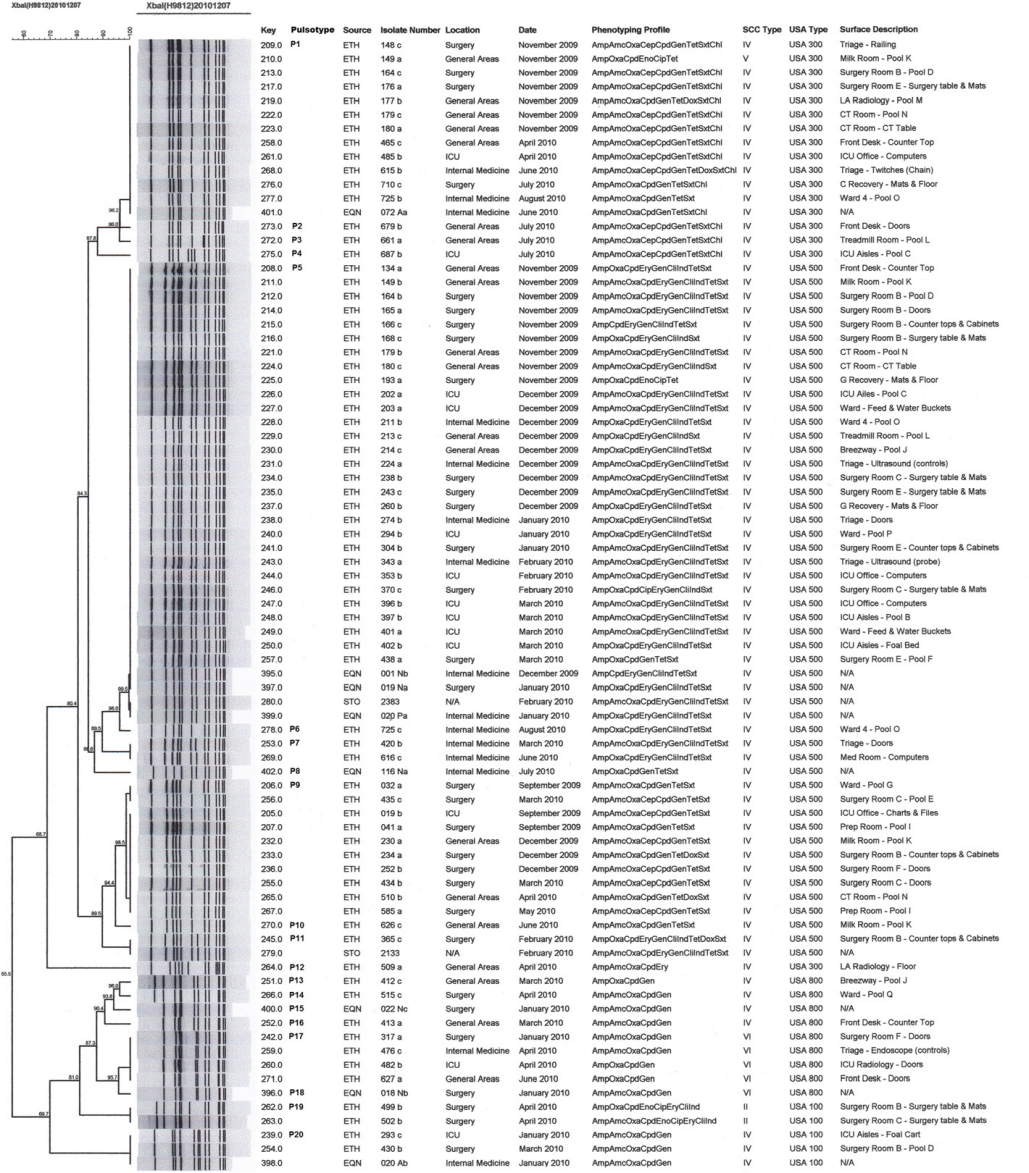
**Dendrogram analysis of environmental and equine MRSA isolates obtained at an equine teaching hospital.** The percent similarity was calculated with Dice coefficients from the PFGE data. Band position tolerance and optimization were set at 1%. ETH: isolate from the environment, EQN: isolate from incoming equine patients, STO: isolate from equine clinical case, ICU: Intensive Care Unit, AMK: Amikacin, AMP: Ampicillin, AMC: Amoxicillin with Clavulanic Acid, CPD: Cefpodoxime, CEP: Cephalothin, CHL: Chloramphenicol, CIP: Ciprofloxacin, CLI: Clindamycin, DOX: Doxycycline, ENO: Enrofloxacin, ERY: Erythromycin, GEN: Gentamicin, OXA: Oxacillin, SXT: Sulfamethoxazole with Trimethoprim, TET: Tetracycline.

Genotypic characteristics of the 71 environmental isolates can be found in Table 2. Over 90% of the isolates carried SCC*mec* type IV and 62.0% were classified as USA500. Analysis of the dendrogram constructed with only the environmental isolates showed 17 different pulsotypes (P1 – P17) grouped in two clusters; cluster 1 included 83.1% (59/71) of the isolates. Dendrogram analysis of both environmental and equine isolates showed that three specific pulsotypes (P5, P1 and P9) represented the majority of the isolates throughout the year, with 29, 12 and 10 environmental isolates each respectively (Figure 2). The most prevalent pulsotype (P5) included isolates characterized as USA500, and was present in the hospital environment for five consecutive months (November 2009 to March 2010). During this period, P5 was evenly distributed on animal and human contact surfaces, and was found in all the hospital services included in the surveillance; in some cases contaminating the same surface for two consecutive months. In total, 7 and 5 pulsotypes were classified as USA500 and USA300 respectively.

**Table 2:** Molecular characterization of environmental MRSA isolates obtained from an equine teaching hospital

**Environment**	By surface* (Total N = 66)		**By isolate (Total N = 71)**	
	**MRSA**	**%**	**MRSA**	**%**
***SCC*** **mec *typing***				
Type II	2/66	3.0%	2/71	2.8%
Type IV	60/66	90.9%	64/71	90.1%
Type V	1/66	1.5%	1/71	1.4%
Type VI	4/66	6.1%	4/71	5.6%
***PFGE***				
USA 100	4/66	6.1%	4/71	5.6%
USA 300	16/66	24.2%	16/71	22.5%
USA 500	44/66	66.7%	44/71	62.0%
USA 800	7/66	10.6%	7/71	9.9%

*Some surfaces were contaminated with two different isolates at the moment they were sampled; therefore, the sum of surfaces by SCC*mec* and PFGE will add up to more than 66.

Phenotypically, 21 distinct antimicrobial susceptibility profiles were identified. Based on the combined results of the phenotypic profile, SCC*mec* type and PFGE pulsotype of the 71 isolates, 34 unique strains (combinations) were found. Over 70% (25/34) of these strains were considered MDR MRSA. Besides beta-lactam resistance, 85.3% (29/34) of the strains were resistant to Gentamicin, 67.3% (23/34) were resistant to Sulfamethoxazole with Trimethoprim and 64.7% (22/34) were resistant to Tetracycline. In addition, all Clindamycin resistant strains (35.3%, 12/34) possessed inducible and not a constitutive resistance. All of the strains (100%) were susceptible to Vancomycin and Amikacin.

### MRSA environmental contamination by type of contact surface (human vs. animal)

MRSA monthly prevalence and distribution by surface type is presented in Figure 1. Detailed information of MRSA contamination on each contact surface (human and animal) that was sampled during the surveillance is described in Table 3. During the surveillance, MRSA contamination of human (9.7%, 43/444) and animal (7.1%, 23/326) contact surfaces were very alike (Tables 3 and 4) (*P* = 0.19). Not including pool samples, the most common human contact surface contaminated with MRSA was the computers (16.7%, 4/24). Among the animal contact surfaces, the feed and water buckets (16.7%, 2/12), followed very closely by surgery tables and mats (15.6%, 7/45), were the most contaminated.

**Table 3:** Prevalence of MRSA contamination on human and animal contact surfaces at an equine teaching hospital

**Contact surface**	**MRSA/samples collected**	**Prevalence per surface**
***Human contact surfaces***		
Computers (keyboards and mouse)^1^	4/24	16.7%
Counter tops & cabinets	6/58	10.3%
Doors	10/103	9.7%
Charts/files	1/12	8.3%
Endoscope (Controls)	1/12	8.3%
Ultrasound (Controls)	1/12	8.3%
Carts	0/22	0.0%
Counter tops, cabinets & sink^2^	0/12	0.0%
Hoist Controls	0/12	0.0%
Twitches (Handle)	0/12	0.0%
Pool samples J and K^3^	5/24	20.8%
Pool samples C, G, H, I, L, M, and N^4^	10/83	12.0%
Pool samples A, B, D, E, and F^5^	5/58	8.6%
**Total**	**43/444**	**9.7%**
***Animal contact surfaces***		
Feed & water buckets	2/12	16.7%
Surgery table & mats	7/45	15.6%
CT table	1/12	8.3%
Floor	1/12	8.3%
Foal bed	1/12	8.3%
Foal cart	1/12	8.3%
Railing	1/12	8.3%
Twitches (chain)	1/12	8.3%
Ultrasound (probe)	1/12	8.3%
Mats & floor^6^	3/47	6.4%
Door, wall & floor^2^	0/12	0.0%
Endoscope	0/12	0.0%
Floor, drain & stall mat^7^	0/12	0.0%
Foal watch mats	0/12	0.0%
Stocks	0/12	0.0%
Pool samples O, P, and Q^8^	4/36	11.1%
**Total**	**23/326**	**7.1%**

^1^Two computers (Keyboards and mouse) were sample as a pool.

^2^Surfaces sampled from four isolation stalls as a pool.

^3^Pool samples J and K included: Light switches, counter tops, computers (keyboards and mouse), phone and doors within the same area. These Pool samples were collected from General Areas.

^4^Pool samples C, G, H, I, L, M, and N included: Counter tops, cabinets and other contact surfaces within the same area. These Pool samples were collected from Intensive Care Unit, Surgery and General Areas.

^5^Pool samples A, B, D, E, and F included: Light switches, phone, oxygen and suction valves, radio, microwave, lamps and medical carts within the same area. These Pool samples were collected from Internal Medicine, Intensive Care Unit and Surgery.

^6^Samples collected in the surgery recovery rooms.

^7^Surfaces sampled in three stalls as a pool.

^8^Pool samples O, P, and Q included: Halters, hay bags, and muzzles (3 of each) within the same ward. These Pool samples were collected from Internal Medicine, Intensive Care Unit and Surgery.

**Table 4:** Overall prevalence of MRSA contamination distributed by services at an equine teaching hospital

**Service**	**Human contact MRSA/samples collected**	**Animal contact MRSA/samples collected**	**Total contact MRSA/samples collected**
***Internal medicine***	5/84 (6.0%)	5/103 (4.9%)	10/187 (5.3%)
***Intensive care unit***	8/94 (8.5%)	5/72 (6.9%)	13/166 (7.8%)
***Surgery***	15/172 (8.7%)	11/115 (9.6%)	26/287 (9.1%)
***General***	15/94 (16.0%)	2/36 (5.6%)	17/130 (13.1%)
**Total**	43/444 (9.7%)	23/326 (7.1%)	66/770 (8.6%)

Genotypically, 13 and 7 distinct pulsotypes were found on human and animal contact surfaces respectively; only 3 pulsotypes (P5, P1 and P20) were present on both types of surfaces. It is important to highlight that P5 and P1 were the two most prevalent clones at the hospital and belong to the same clonal cluster (Figure 2). Conversely, the third most prevalent pulsotype (P9) was exclusively found on human contact surfaces. Considering P5 alone, 34.8% (16/46) of the MRSA isolates from human contact surfaces and 52.0% (13/25) of the isolates from animal contact surfaces were contaminated by this clone, but no significant difference was detected (*P* = 0.15). In addition, P5 was found contaminating the same surface for 2 consecutive months on two separate occasions. This was the case on the mats and floors of a surgery recovery room (Nov-Dec) and the computers at the ICU office (Feb-Mar). Conversely, it was also observed that six surfaces were contaminated for 2-3 consecutive months with different pulsotypes, showing a constant reintroduction of strains. Some of these surfaces were the front desk doors, tables and mats, counter tops and cabinets, and computers. It is important to notice that 5 out of the six surfaces continuously contaminated with new strains were human contact surfaces. Pool sample K (which included light switches, counter tops, computers keyboards and mouse, phone and doors of the milk room) was also found positive for 2 consecutive months with 2 different pulsotypes. However, since these samples were collected from several surfaces as a pool, we cannot conclude that it was indeed the same surface that was always contaminated.

### MRSA environmental contamination by hospital service

The overall MRSA contamination in each hospital service and the detailed prevalence by type of contact surface is described in Table 4. There was not a difference among the prevalences of the 4 services (*P* = 0.109). However, it is important to highlight that the same surfaces were not always sampled in each service (e.g. not all the services had an endoscope or a foal bed) (Table 1). Furthermore, the level of exposure (to human or animal contact) varies from surface to surface. Hence, any comparisons among services must be done with care. In any case, the General Areas of the hospital had the most diverse contamination, with 11 different pulsotypes found cycling during the year surveillance. The most prevalent pulsotype (P5) was present contaminating different surfaces from 2 to 5 consecutive months: General Areas for 2 months, Internal Medicine 3 months, ICU 4 months, and Surgery 5 months.

### MRSA environmental contamination by season

MRSA environmental contamination was detected in the equine hospital in eleven out of twelve months that were sampled. The months of November and December had the greatest overall (human and animal contact surfaces) MRSA prevalence, as 18.5% (24/66) of the surfaces sampled each month were contaminated (Figure 1). The 28 isolates obtained during these 2 months, accounted for 39.4% (28/71) of all the MRSA isolates collected during the surveillance. When analyzing by season, the prevalence of MRSA from Jan-Mar was 10.2% (20/196), Apr-Jun 7.2% (14/194), Jul-Sep 4.3% (8/184) and Oct-Dec 12.2% (24/196). The analysis showed that the season of the year was associated with the prevalence of MRSA (*P* = 0.034) with the highest prevalence during the fall months (October to December). These results were confirmed by the logistic regression models which showed a significant difference between the summer (lowest contamination) and the fall (*P* = 0.04).

### Prevalence and characteristics of MRSA equine isolates

A total of 120 incoming horses were sampled in parallel to the active environmental surveillance, and 5.8% (7/120) were MRSA positive in at least one anatomical location. Epidemiological data regarding these horses will not be discussed in this manuscript. The anatomical location that most frequently tested positive for MRSA was the nares (71.4%, 5/7) followed by the armpits (28.6%, 2/7) and the perianal area (14.3%, 1/7). None of the skin lesions sampled were positive for MRSA. Only one horse was positive in two anatomical locations (armpit and perianal); therefore, both isolates (one from each location) were included for further phenotypic and genotypic characterization, resulting in a total of 8 equine MRSA isolates to be analyzed in this study.

Genotypically, 87.5% (7/8) of the isolates were classified as SCC*mec* type IV, and 12.5% (1/8) as type VI. Fifty percent (4/8) were characterized as USA500, 25.0% (2/8) as USA800, 12.5% (1/8) as USA300 and 12.5% (1/8) as USA100. Dendrogram analysis of only the equine isolates (not shown here) revealed 6 different pulsotypes distributed in two major clusters with only 59.2% similarity among them; one of the isolates was not related to either cluster. The two isolates obtained from different anatomical locations on the same horse were classified as different pulsotypes (one of them was the isolate not included in any cluster), which is indicative that a horse could be potentially colonized with more than one strain. When compared against environmental isolates, 5/8 equine isolates matched with three environmental pulsotypes (P1, P5 and P20) circulating in the hospital (Figure 2). The other 3 equine isolates were characterized as unique pulsotypes that were never detected or previously seen in the environment of the hospital.

Of the 8 equine MRSA isolates, 6 unique strains were identified based on the combined results of phenotypic profile, SCC*mec* type and PFGE pulsotype. Fifty percent of the strains (3/6) were classified as MDR. Similarly to environmental strains, the equine strains were resistant to Gentamicin (100%, 6/6), Sulfamethoxazole with Trimethoprim (50.0%, 3/6) and Tetracycline (50.0%, 3/6). Only one strain was found to have inducible resistance to Clindamycin. All the equine strains (100%) were susceptible to Vancomycin, Amikacin, Ciprofloxacin, Enrofloxacin and Doxycycline.

### Molecular comparison of MRSA isolates from clinical cases and the environment

In February 2010, two equine patients were admitted to the hospital for colic and developed MRSA post-operative infections in their surgical incisions. Neither one of these horses were screened for MRSA at their arrival to the hospital. In both cases the interventions were performed in the same surgery suite (Room B), which was sampled during the monthly environmental surveillance on February 10^th^. Isolates obtained from these two patients were compared against the environmental isolates using PGFE band patterns and dendrogram analysis. The first patient (patient A) was admitted to the hospital on February 4^th^. This patient had two surgical procedures on February 4^th^ and February 9^th^; both surgeries were performed in the same surgery suite. On February 18^th^ the patient was diagnosed with a MRSA infection of its surgical incision. After molecular analysis, the MRSA isolate from this patient was determined to be identical to one of the most prevalent environmental pulsotypes (P5) circulating in the hospital during that sampling period as well as in previous months (Figure 2). The second patient (patient B) was admitted to the hospital on February 8^th^ and underwent surgical procedures on February 8^th^ and February 18^th^. On February 25^th^, a specimen collected from the patient’s surgical incision was positive for MRSA. When compared against the environmental isolates, the equine isolate matched with one pulsotype (P11) that was only seen in the hospital environment one time during the whole study (Figure 2). This unique pulsotype was found on February 10^th^ on the counter tops and cabinets from the suite where the surgery took place, and it was never seen before or after this event.

## Discussion

No previous reports have been published on estimating the prevalence and distribution of MRSA in an equine veterinary hospital environment over an extended period of time, and regardless of the presence of patients with MRSA infections. In consequence, little is known about the potential that environmental surfaces may play as a source of infections for patients and hospital personnel. Since MRSA can affect humans and horses, the presence of this zoonotic pathogen in the environment may increase the occupational and nosocomial risk for infection. We demonstrated that MRSA was present on 8.6% of the environmental surfaces sampled throughout the yearlong surveillance. Previously only one cross-sectional study has been performed during a non-outbreak period finding 4.3% MRSA contamination [21]. Other studies have found environmental contamination ranging from 9.6% to 52.7% [16,17,22]. However, it is important to highlight that these latter results were obtained when sampling the hospital environment during or after MRSA outbreaks involving equine patients. Our results clearly demonstrate that MRSA is frequently present contaminating the environment throughout the year, not necessarily associated with outbreaks.

When the patterns of MRSA environmental contamination at the hospital were analyzed three important scenarios were noticed: constant introduction and reintroduction of strains, circulation (movement) of clones throughout the hospital services, and maintenance (survival) of strains overtime in the environment.

In the first scenario, we noted a continuous introduction of MRSA strains into the hospital, where “new” MRSA clones not previously observed in the surveillance were found on several environmental contact surfaces. Of the 17 pulsotypes that were detected in the environment, 10 of them were observed only once in the 12 months study period. For example, P16 and P4 were only detected in March and July, respectively, and none of them was seen before or after that date (Figure 2). Also, reintroduction was observed when a clone was initially detected in the environment, and then disappeared for several months before showing up again in a different area or on another surface later on. For example, P17 was observed in January (surgery) for the first time, it then disappeared for several months, to be detected again in April (ICU and internal medicine), and later in June (general areas) (Figure 2). Similar patterns of reintroduction were noticed with other pulsotypes. This scenario of introduction and reintroduction of this pathogen into the environment highlights the importance of performing continuous surveillance and monitoring to identify the most common strains circulating in the hospital as well as identifying new strains that could represent additional risk.

The second scenario associated with the circulation or movement of MRSA strains across the hospital services, was observed when a unique pulsotype appeared in one specific area or on one surface and was then detected in the continuing months on other surfaces and/or in other sections. This was the case of P5, which was first detected on surfaces from the General Areas and Surgery in November. Then, during the next 4 months, P5 circulated through all the services sampled and by March was mostly detected in the ICU service. After March this pulsotype was never detected again in the environment. Similar scenarios were observed with other pulsotypes, which were detected simultaneously in different services and on surfaces but for shorter periods of time (three consecutive months). These examples reveal the circulation of MRSA clones throughout different areas of the hospital, perhaps carried by hospital personnel, as this has been described in other health care settings [28,29]. It is also possible that this situation could occur due to the constant reintroduction of the same clone into the hospital environment. However, we cannot reject the possibility of both scenarios (reintroduction and circulation) happening in parallel to each other. Based on these facts, it is highly recommended to emphasize the importance of biosecurity and personal hygiene practices of the hospital personnel, primarily their compliance with hand washing. Contaminated hands and/or the gloves of healthcare workers might be involved in the transmission of nosocomial pathogens like MRSA [28,30]. On the other hand, it cannot be denied that the equine patients (colonized or infected with MRSA) may have played a role in the movement of this bacterium across the hospital; this issue was not assessed in this study, but it has been previously suggested [17]. For this reason, the use of biocontainment measures, especially when handling suspicious and/or confirmed MRSA cases, should be strictly implemented.

The third scenario observed was associated with the maintenance of MRSA strains overtime in the hospital environment. We detected contamination of the same surface with the same unique pulsotype for two consecutive months. This was the case for the computers of the ICU office, and the mats and floor of a recovery room. We reported similar results in a small animal hospital, where the gurneys used to move patients were found contaminated with the same MRSA pulsotype over 3 consecutive months [23]. The presence overtime of the same strain on a specific surface is not surprising, as MRSA has been reported to survive on inanimate surfaces for up to 7 months [20,30]. In any case, the fact that a MRSA clone was able to survive on a specific surface for up to 2 months in our study, suggest that insufficient cleaning and disinfection protocols are in use, highlighting the importance of identifying and targeting environmental surfaces that are continuously contaminated to be more rigorously addressed.

We identified several surfaces that were contaminated multiple times throughout the year, either with different pulsotypes or in some cases with the same clone. Among the most commonly contaminated human contact surfaces were the computers (16.7%), counter tops and cabinets (10.3%) and doors (9.7%); and among the animal contact surfaces the feed and water buckets (16.7%) and surgery tables and mats (15.6%). All of these surfaces are in contact with many individuals, both human and animal, and thus have a high likelihood of contamination with MRSA. These surfaces might be considered “hot spots” for MRSA contamination and could potentially become sources of nosocomial infections; therefore, they must be included in any cleaning and disinfection program. Since each practice/hospital is unique, each one should document baseline contamination with MRSA and identify “hot spot surfaces” to target for cleaning and disinfection.

Phylogenetic analysis showed very little diversity of the MRSA strains circulating in the equine hospital (83% of the isolates had ≥ 80% similarity). Furthermore, the molecular analysis showed that the majority of our isolates carried SCC*mec* type IV (90.1%) and were identified as USA500 (62.0%). This PFGE clone has been frequently reported in horses as an endogenous MRSA strain and has been classified as a nosocomial or hospital-acquire MRSA (HA-MRSA) [31,32]. These results provide further evidence that among the constellation of MRSA strains circulating in humans, certain strains are more likely to circulate in horses, their environments and their human contacts as has been previously suggested [4,6,32,33]. In contrast, other strains classified in this study as USA100, USA300 and USA800 are clones frequently reported in the US human population, either as a cause of nosocomial infections (HA-MRSA, USA100 and USA800) or present in the general population (also known as community-acquired MRSA [CA-MRSA], USA300) [34,35]. The presence of these strains on different surfaces and areas across the hospital highlights the role that humans (personnel and/or clients) could possible play in the contamination of the hospital environment and as a source of nosocomial infections for patients.

We could not definitively identify the source of MRSA infection in the two post-surgical cases in our study. The isolate from patient A was indistinguishable from the P5 isolate present in the hospital environment. Since P5 was present in the hospital 3 months before and 1 month after patient A’s isolate was obtained, we could not establish a temporal relationship. However, the likelihood of acquiring a nosocomial infection with the most common strain circulating the hospital at the time of the surgery is a possibility that could not be discarded. Conversely, the isolate obtained from patient B was indistinguishable from P11, a pulsotype found in the hospital environment only once during the 12 month surveillance. Two possible scenarios can be described. First, it is possible that patient B developed a nosocomial MRSA infection due to indirect contact with contaminated surfaces present in the surgery room during the second intervention (exogenous sources). Second, it is also possible that patient B was already colonized with this particular strain upon arrival to the hospital (endogenous source). Unfortunately, due to the design of the study, we cannot be certain of the source of the MRSA infection in patient B; especially due to the fact that only a representative portion of the hospital environment was sampled. Therefore, it is possible that P11 could have been in other areas/surfaces of the hospital that were not included in this study.

Confirming the actual source of the MRSA strains present in the hospital environment will require further studies to include the sampling of all three components (human, animal and environment) involved in the ecology of this pathogen. Results of the present study suggests that hospital personnel may be an important source of MRSA, since the majority (5/6) of the surfaces that were frequently contaminated for 2-3 consecutive month were human contact surfaces. Also there was a higher diversity of clones detected on human contact surfaces (13 clones) than on animal contact surfaces (7 clones). Nonetheless, horses may have still been the primary source responsible for the contamination detected among the animal contact surfaces. This scenario is even more probable if we consider that 5.8% of the incoming horses were found positive for MRSA upon arriving to the hospital with strains that were also detected in the environment. Moreover, it is important for other veterinary hospitals to consider this possibility, especially since higher prevalences of MRSA in incoming equine patients have been reported in other countries [36]. In any case, since the colonization status of all incoming equine patients and the hospital personnel was not established during the study period, we can only speculate that both groups were involved in the introduction of MRSA to the GEC.

Lastly, we acknowledge that the design used for this environmental surveillance had limitations. First, multiple surfaces were sampled as a pool when necessary due to financial constraints, interfering with our ability to determine in some cases which particular surface was positive. Yet, pooled sampling is a good alternative during routine surveillance as it allows us to establish if a particular area/room is contaminated with MRSA. Second, it is important to recognize that differences among veterinary hospitals in the US and other countries may reduce the ability to extrapolate from the results presented. Nonetheless, this study left no doubt that MRSA is present and circulating in an equine veterinary environment, and these findings can be used during the development of surveillance programs and cleaning and disinfection control plans in other institutions.

In conclusion, this is the first report of a yearlong environmental surveillance performed at a large equine hospital, and it was confirmed that MRSA is present on different contact surfaces during a non outbreak period. We observed that different MRSA strains were not only constantly introduced and/or reintroduced into the hospital, but they were also moved among and maintained in the environmental surfaces of different sections of the hospital. The presence of MRSA in all but one of the 12 months of the surveillance, the detection among human and animal contact surfaces across multiple services, and the presence of MDR profiles are all causes of concern from the point of view of occupational safety as well as control and prevention of nosocomial infections. These findings highlight the necessity of maintaining effective cleaning and disinfection protocols at all times, as well as the importance of performing continuous surveillance to identify strains circulating the hospital as well as the surfaces that could act as “hot spots” and reservoirs for this zoonotic and nosocomial pathogen.

## Abbreviations

MRSA: Methicillin-resistant *Staphylococcus aureus*; GEC: Galbreath Equine Center at The Ohio State University Veterinary Medical Center; PFGE: Pulsed-field gel electrophoresis; MDR: Multidrug resistant.

## Competing interests

The authors declare that they have no competing interests.

## Authors’ contributions

Experimental design and planning: CK and AEH. Collection of environmental samples: JVB, JM, RCN. Collection of equine samples: MPS. Laboratory processing: JVB, JM, RCN. Data processing and statistical analysis: JVB and JM. Drafting of the manuscript: JVB, JM and AEH. Critical revision of the manuscript: JVB, JM, MPS, RCN, CK and AEH. All authors read and approved the manuscript.
